# Strain Dependent Evolution of Microstructure and Texture During Cold Rolling of Ferritic Stainless Steel: Experiments and Visco-Plastic Self-Consistent Modeling

**DOI:** 10.3390/ma18050995

**Published:** 2025-02-24

**Authors:** Jibin Pei, Shilong Wei, Qing Zhang, Xiufang Ji, Chi Zhang, Luyang Miao

**Affiliations:** 1 School of Railway Locomotive and Vehicle, Jilin Railway Technology College, Jilin 132299, China; peijb76@163.com (J.P.); yyq_858962@163.com (S.W.); 2 Inner Mongolia North Heavy Industries Group Corp. Ltd., Baotou 014033, China; zhangqing8258@163.com (Q.Z.); 15024795641@163.com (X.J.); 3 School of Materials Science and Engineering, Dalian University of Technology, Dalian 116024, China; luyangmiao22@163.com

**Keywords:** ferritic stainless steel, cold rolling, microstructure, texture, VPSC

## Abstract

In the present work, the microstructure and texture evolution of ferritic stainless steel during unidirectional cold rolling were investigated, and the Visco-Plastic Self-Consistent (VPSC) polycrystal model was used for the simulation of texture during cold rolling. Comparison of different interaction models was made to obtain a model that better reproduces the texture evolution of ferritic stainless steels. The as-received hot-rolled samples were unidirectionally cold rolled in a laboratory rolling mill, and the thickness was reduced by 30%, 60% and 80%. Electron backscatter diffraction (EBSD) was used to observe the microstructure evolution and texture evolution, and micro-hardness was used to evaluate the work hardening of the sample. The important feature of the microstructure was the presence of shear bands (SBs), the frequency of which increased with the increase in cold-rolling reduction and was found to be orientation dependent. We found that the geometrically necessary dislocation (GND) density increased with cold-rolling reduction in accord with Ashby’s theory of work hardening, and higher GND density accumulates near the grain boundary. The grain fragmentation, Goss texture distribution and orientation gradient were found to be orientation dependent. The cold-rolled texture was composed of strong α-fiber and weak γ-fiber. The relative plastic compliance of grain and the homogeneous effective medium (HEM) were explored. The tangent interaction model was found to match reasonably well with the experimental texture. This work has great significance for achieving online monitoring of the texture of ferritic stainless steel under different industrial production processes and enhancing the intelligence level of ferritic stainless steel production process.

## 1. Introduction

Ferritic stainless steel [1] has a lower manufacturing cost, smaller thermal expansion coefficient and excellent resistance to pitting and stress corrosion [2]. Hence it has a good application prospect. Concerning on properties, ferritic stainless steel can be widely used for automotive structural parts, kitchen equipment and key industrial components. The r value determines the formability of ferritic stainless steel. However, the r value (1.42) of ferritic stainless steel [3] is lower than the r value (2.43) of interstitial-free (IF) steel [4]. Therefore, it is significant to carry out detailed research to further improve the formability of ferritic stainless steel.

The formability of ferritic stainless steel depends on the {111}//ND recrystallization texture, also known as γ-fiber [5]. Although industrial ferritic stainless-steel sheet is mainly in annealed condition, the recrystallization texture of low carbon steel with severe cold-rolling reduction (75–90%) is thought to be controlled primarily by oriented nucleation [6,7]. During recrystallization, the dominant nucleation site is the γ-fiber grains formed in the short-range and high-energy storage region of γ-fiber grains. It occurs near the grain boundaries and in-grain SBs after cold rolling [8,9]. With the increase of the cold-rolling reduction, the grain size decreases and the grain boundary area increases. Hence, the relative contribution from grain boundary nucleation increases, as does the intensity of the γ-fiber recrystallization texture. The nucleation of cold-rolled recrystallization is not nucleation in the sense of classical thermodynamics, but rather the abnormal growth of the fragmentary structure (cell or sub-grain) of the cold-rolled sample [10]. Therefore, in order to further improve the formability of ferritic stainless steels, it is necessary to understand the SBs, grain boundaries, in-grain fragmentary degree and related orientation changes during cold rolling.

Furthermore, the inhomogeneity of deformation during cold rolling, local force equilibrium and geometrical shape compatibility must be maintained at the grain boundaries. This requirement that results in local phenomena such as accumulation due to strain incompatibility and to changes in elastic and plastic properties induced by elastic anisotropy and a change in grain orientation [11]. According to the theory of Ashby [12], it can be divided into statistically stored dislocation (SSD) or geometrically necessary dislocation (GND) from the perspective of mechanics. In the uneven deformation of polycrystalline materials, GND is used to connect the deformation of individual grains in the polycrystalline to ensure the continuity of the entire grain boundary. Generally, compared with the inside of the grain, the grain boundary region shows a larger orientation gradient and a higher GND density. The high GND density and orientation gradient in the grain boundary region make them the source of grain nucleation during recrystallization. Therefore, the microstructure of the grain boundary region plays an important role in determining the properties of ferritic stainless steel. The strong and uniform γ-fiber is beneficial to improve the deep-drawing performance of ferritic stainless steels, while the significant Goss components are detrimental to formability. The recrystallization texture of Goss mainly comes from the cold-rolled {111}<112> grain with SBs [13]. However, the fragmentation of Goss grains with strain is not completely clear.

In recent years, due to the gradual development of crystal plasticity models, the ability to predict the texture of cold-rolled ferritic stainless steel has been greatly improved. The original full constraints (FC) Taylor model assumes that the strain in each grain is equal to the externally applied strain, and an independent slip system in each grain needs to be activated. However, this constraint is too strong and tends to predict the wrong texture, as reported in [14]. Houtte et al. [15,16] developed the Lamel model, which appropriately relaxes some components of the constraint (L_13_ and L_23_ shear). Although it can predict the well-deformed texture of steel with 70% reduction [15], the model still applies uniform strain to each grain like the FC model. With the further development of the FC model, the FC model has variations such as the relaxed constraints method (RC) and the self-consistent (SC) model. Toth et al. [17] used RC and SC models to compare the pole figures of ferritic stainless steel with 90% reductions. Engler et al. [18] developed the grain-interaction (GIA) model, which can predict the texture of aluminum under high deformation.

Regarding the visco-plastic self-consistent (VPSC) model [19], it has been widely used to predict the deformation texture. The model assumes that each grain can be regarded as an ellipsoidal inclusion embedded in a homogeneous effective medium (HEM) with average properties of the material. It does not specify the strain component in the grain and takes into account the influence of the grain shape. The rotation of all grains in this model is not directly constrained by specific neighboring grains, but only constrained by the HEM, causing all grains with the same initial orientation to be reoriented in the same way. VPSC allows modification of the compliance between the grain and the HEM, and compliance has an important influence on the prediction of deformation texture [20]. Després et al. [21] studied initial single equiaxed grains and single pancaked grains; the texture prediction using the Affine VPSC model was in good agreement with measured texture at different cold-rolling reductions. Takajo et al. [22] used Taylor, Secant, Tangent and RDC models to predict the deformation texture of ultra-low carbon steels with different reductions. They showed that using different interaction models for different deformations gave a reasonably good predictions. Gupta et al. [23] used FC, Affine, n^eff^ = 10, Secant and Tangent models to predict the deformation texture of β-titanium in the multi-step cross rolling process and showed that the FC model can better reproduce the deformation texture. In Zhang et al. [24], a modified Voce (ME-Voce) model is proposed, based on the extended Voce hardening model. The results show the VPSC embedded in the ME-Voce can reasonably predict the mechanical response under different deformation conditions. However, the above research has not used VPSC with different interaction models to simulated cold-rolling texture when the as-received ferritic stainless steel hot-rolled samples are composed with hybrid grains i.e., equiaxed grain and pancaked grain.

In this work, we studied the microstructure of cold-rolled ferritic stainless steel with different reductions and used the different interaction models of VPSC to predict the texture. Experimentation reveals the influence of different reductions on the microstructure and texture. Quantitative and qualitative comparisons between experimental measurements and predictions from the VPSC model and the influence of grain and HEM compliance on the prediction of rolling texture were analyzed. A detailed understanding of the cold-rolling process of ferritic stainless steel should be an aspect of optimizing the formability of ferritic stainless steel.

## 2. Experimental Methodology & Model Description

### 2.1. Cold Rolling, Microstructure and Texture Characterization

**Figure 1:**
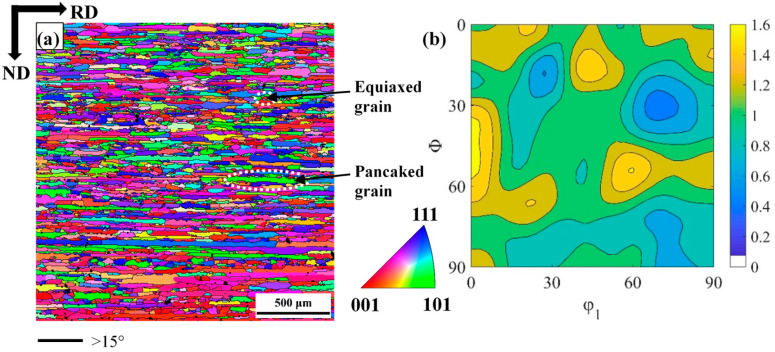
(**a**) Inverse pole figure (IPF) map of as-received sample; (**b**) φ_2_ = 45° section of ODF of as-received sample.

The chemical composition (wt%) of the industrial hot band of single-phase ferritic stainless steel used in this work is given in Table 1. The hot-band thickness is 5.65 mm, and the microstructure was composed of pancaked as well as equiaxed grains, as shown in Figure 1a.

The ferritic stainless-steel sheets were unidirectional cold rolling in the original hot rolling-direction in a laboratory mill (designed in-lab with the diameter of roller being 150 mm) to obtain sheets with different reductions. The reductions were 30%, 60% and 80%, corresponding to thicknesses of 3.96 mm, 2.26 mm and 1.13 mm, respectively.

For all samples, the microstructure characterization was performed on rolling direction (RD)–normal direction (ND) plane. The micro-hardness (HV, AutoVicker 1000AF Pro) of the deformed sample was measured with a load of 25 g and a remain load force of 15 s. Take 10 readings for each condition to obtain the average hardness value and its standard deviation. The scanning electron microscope (SEM, JSM-IT800, JEOL Ltd., Tokyo, Japan) equipped with an electron backscatter diffraction (EBSD, Oxford Symmetry2, accelerating voltage: 20 kV, tilt angle: 70°, step size: 1.5 μm) system was used to characterization the microstructure and texture. The samples were prepared by standard mechanical polishing to 1.5 μm diamond, and then, using DC power (MS-305DS, voltage: 30 V, time: 20 s), electropolished in an 8% perchloric acid-92% alcohol solution. The observation was conducted at the central fifth of the thickness of the sample, where the deformation can be well-approximated as plane strain compression [25]. The texture was measured using more than 110,000 points in the RD-ND plane, which span at least 10,000 grains (see [26]), which will provide results nearly consistent with X-ray diffractometer (XRD). The orientation distribution function (ODF) was calculated using MTEX and the assumption of orthotropic samples symmetric. ODFs were drawn in the φ_2_ = 45° Euler space, as it contains most of the texture information [25]. The ODF obtained from the simulation was calculated using the same settings.

### 2.2. Model Description

The simulation was carried out using the open source VPSC code from Los Alamos National Laboratory. In the VPSC model [19], each representative orientation is modeled as an ellipsoidal grain embedded in a homogeneous equivalent medium (HEM) with averaged aggregate properties. The visco-plastic constitutive behavior that relates the stress σ, and the strain rate $\dot{\varepsilon }$, in a given grain inclusion is described by means of the non-linear rate-sensitivity equation:

(1) $${\dot{\varepsilon }}^{c}=\sum_{s=1}^{N}m^{s}{\dot{\gamma }}^{s,c}$$

${\dot{\gamma }}^{s,c}$ can be formulated as follows:

(2) $${\dot{\gamma }}^{s,c}={\dot{\gamma }}_{0}^{s}\frac{\left|m^{s}:\sigma^{c}\right|}{\tau^{s,c}}\mathrm{sgn}(m^{s}:\sigma^{c})$$

In the above expression, m, $\dot{\gamma }$, ${\dot{\gamma }}_{0}$ and τ, respectively denote the Schmid tensor, the shear rate, normalization factor of shear rate and the critical resolved shear stress (CRSS). The parameters c, s, N and n are, respectively, the ellipsoidal grain, the slip system, the total number of slip systems and the flow rule exponent.

In the present work, we considered all three slip modes common to body-centered cubic (BCC) crystals [27,28]: 12 slip systems in {111}<110>, 12 slip systems in {112}<111> and 24 slip systems in {123}<111>. The CRSS is characterized by the extended Voce hardening law that an evolution of the threshold stress with accumulated shear strain in each grain of the form

(3) $${\hat{\tau }}^{s}=\tau_{0}^{s}+(\tau_{1}^{s}+\theta_{1}^{s}\Gamma )(1-\exp(-\Gamma \left|\frac{\theta_{0}^{s}}{\tau_{1}^{s}}\right|))$$

where

(4) $$\Gamma =\sum_{s}\Delta \gamma^{s}$$

is the accumulated shear in the grain; $\tau_{0}$, $\theta_{0}$, $\theta_{1}$ and $\left(\tau_{0}+\tau_{1}\right)$ are the initial CRSS, the initial hardening rate, the asymptotic hardening rate and the back-extrapolated CRSS.

The increment in the threshold stress of a system due to shear activity $\Delta \gamma^{s\prime }$ in the grain systems is expressed as follows:

(5) $$\Delta \tau^{s}=\frac{d{\hat{\tau }}^{s}}{d\Gamma }\sum_{s\prime }h^{ss\prime }\Delta \gamma^{s\prime }$$

where

(6) $$\frac{d{\hat{\tau }}^{s}}{d\Gamma }=\left[\theta_{1}+(\left|\frac{\theta_{0}}{\tau_{1}}\right|\tau_{1}-\theta_{0})\exp(-\Gamma \left|\frac{\theta_{0}}{\tau_{1}}\right|)+\left|\frac{\theta_{0}}{\tau_{1}}\right|\theta_{1}\Gamma \exp(-\Gamma \left|\frac{\theta_{0}}{\tau_{1}}\right|)\right]$$

The VPSC model treats a polycrystalline material by the representative inclusion and the HEM interact to a degree that scale with relative stiffness of the HEM and the ellipsoidal grain. Using the Eshelby’s equivalent inclusion approach [29], the following interaction equation linking the grain’s stress and strain rate, $\sigma^{c}$ and ${\dot{\varepsilon }}^{c}$, with mean stress and strain rate, $\bar{\sigma }$ and $\bar{\dot{\varepsilon }}$, can be derived:

(7) $$\left({\dot{\varepsilon }}^{c}-\bar{\dot{\varepsilon }})=-{\tilde{M}}^{c}(\sigma^{c}-\bar{\sigma }\right)$$

where

(8) $${\tilde{M}}^{c}=n^{eff}{\left(I-E\right)}^{-1}:E:\bar{M}$$

is the accommodation tensor, which is a function of the Eshelby tensor [30] E and macroscopic compliance M.

The n^eff^ is a parameter related to the stiffness of the medium. Its influence on texture prediction is very significant, hence this paper has conducted a detailed study. In general, the inclusion and HEM interaction model [31] are classified according to the value assigned to n^eff^ and are shown in Table 2. Full constraint (FC) model [23,32,33] have null n^eff^ correspond to stiff HEM around grain, and assign the same or nearly the same strain rate in every grain. The tangent model [19,23,34] tends to a wide dispersion strain rata among grains. The larger value of n^eff^ indicates that it is a compliant medium, and each grain is allowed to have a different strain state. In other words, FC interaction model is stiff and the tangent interaction model is compliant. Another intermediate approximation that can give polycrystal responses in between the stiff FC and the compliant tangent approaches is adjustable parameter n^eff^. In this paper, we denoted calculation using n^eff^ = 10 [23,34] as the intermediate model. On the other hand, the affine model [16,35,36] remains upper-bounds for n^eff^ → ∞. Note that when using the Taylor model (n^eff^ = 0), the shape of the Eshelby inclusion does not affect the deformation of the grain, which can be inferred from Equation (8).

In the present calculation of texture development, the velocity gradient was [100 000 00-1] and 0.02 strain increment. Voce hardening parameters [21] ($\tau_{0}$, $\tau_{1}$, $\theta_{0}$, $\theta_{1}$) were set the same for the three slip systems [26,27,37], $\tau_{0}$ = 98.3, $\tau_{1}$ = 128, $\theta_{0}$ = 900, $\theta_{1}$ = 0. The reader is referred to Ref. [38] for their meaning in the extended Voce hardening law. Figure 1b represents the orientation distribution function (ODF) of the hot-band sample. Since the materials include the stabilizing elements (Ti, Nb), the γ-phase grains in the hot-rolled sheets were not recrystallized before being transformed into α-phase grains in the rapid cooling process [39]. The initial texture is discretized on a set of 2000 initial directions, which are imported by the VPSC software (Version 7b) as input.

## 3. Results

### 3.1. Evolution of Microstructure During Cold Rolling

**Figure 2:**
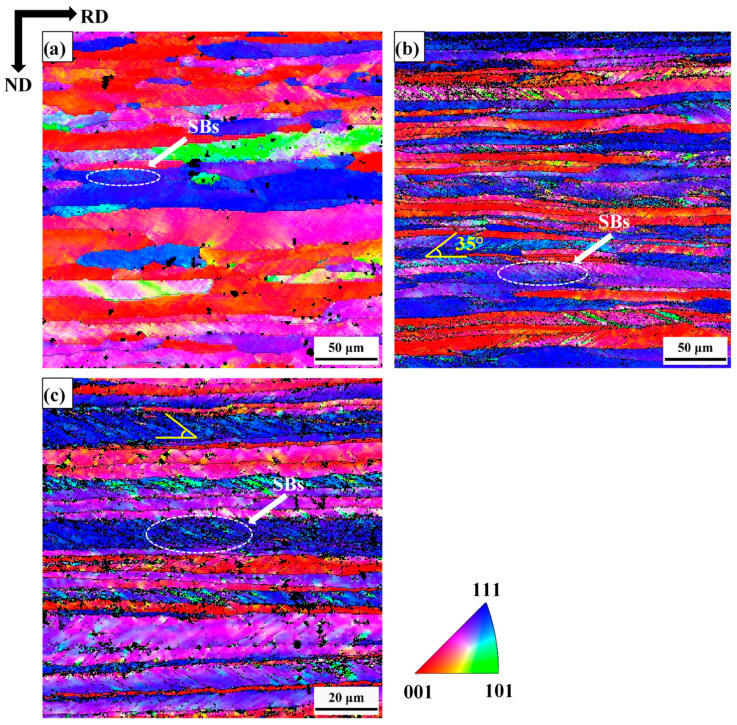
EBSD IPF maps of (**a**) 30% reduction, (**b**) 60% reduction, (**c**) 80% reduction.

**Figure 3:**
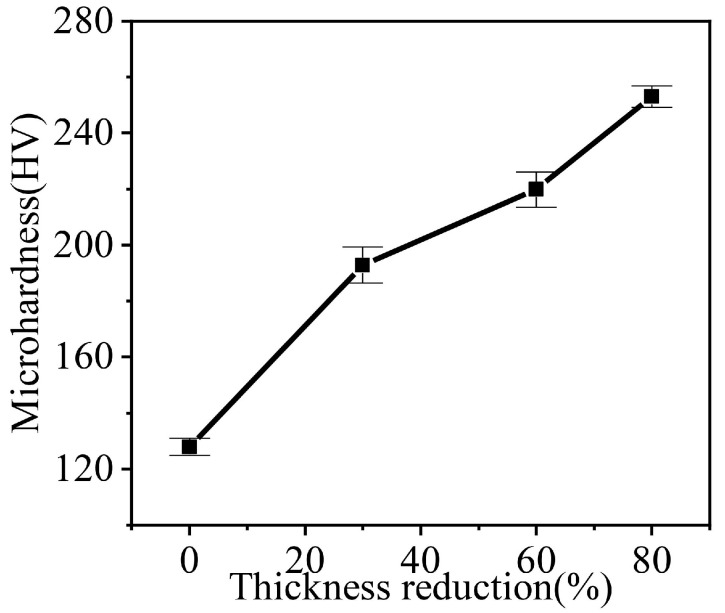
Micro-hardness variation in relation to thickness reduction.

Figure 2 shows inverse pole figure (IPF) maps for 30% reduction, 60% reduction and 80% reduction cold-rolled samples. In the present work, the microstructure band was elongated along the RD. The average thickness of the microstructure band (along with the sample ND) was 25.56 μm, 15.44 μm and 8.13 μm for 30% reduction, 60% reduction and 80% reduction, respectively. As the cold-rolled reduction increases, the average band thickness gradually decreases. The most dominant feature of the deformed microstructure in all the present samples was the presence of in-grain shear bands (SBs). The inclination angle of these SBs was approximately ±35° to the sample RD. SBs density increased with the increase of cold-rolling reduction. At higher cold-rolling reduction (80%), these SBs usually get aligned along the grain boundaries. IPF maps (Figure 2) were composed of grains with SBs and grains without SBs. SBs appear more frequently in grains with γ-fiber orientations than grains with other orientation, i.e., orientation dependence. Marking with a similar appearance has been observed by other authors [9,40,41,42]. It is comparable to SBs reported in the literature for cold-rolled BCC crystals of relatively similar rolling reduction. Figure 3 shows micro-hardness variation in relation to thickness reduction. As the samples thickness decreases, the proportion of SBs as well as micro-hardness were observed to increase. Due to the increase in grain boundary area, SBs and dislocation density, the 80% cold-rolled sample has the highest hardness of 251 HV.

**Figure 4:**
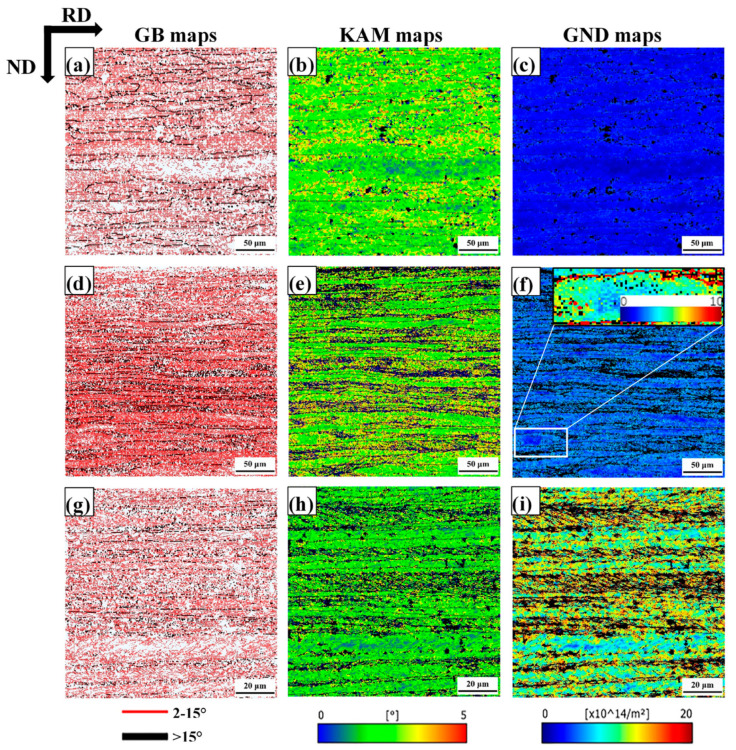
GB, KAM and GND density maps of (**a**–**c**) 30% of thickness reduction, (**d**–**f**) 60% of thickness reduction, (**g**–**i**) 80% of thickness reduction.

Figure 4 shows grain boundary (GB), kernel average misorientation (KAM) and geometrically necessary dislocation (GND) density maps of the samples with 30%, 60% and 80% of thickness reduction, respectively. The distribution of misorientation of cold-rolled plates was analyzed by EBSD (Figure 4a,d,g). Red and black lines denote low angle grain boundaries (LAGBs) and high angle grain boundaries (HAGBs). Figure 4b,e,h shows kernel average misorientation (KAM) maps for the cold-rolled samples with different rolling reductions in thickness. KAM maps are generally considered to highlight areas of higher micro-strain caused by crystal defects, i.e., dislocations [43,44], which can quantitatively characterize the inhomogeneity of the material plastic deformation and defect density distribution. It can be seen that the KAM value of the γ-fiber zone was relatively higher than the KAM value of the α-fiber zone. In the grains, local high micro-strains are concentrated around the grain boundaries, and the KAM value inside the grains shows a gradient change. When the plate was composed of pancaked grains and passes through a narrow plastic deformation zone, the grains will not deform in the same manner throughout their volume [45]. Consequently, a large number of geometrically necessary dislocations (GND) were generated to maintain the continuity of the grains. Those GND will cause large misorientation within the grains and accelerate the splitting of the grains into sub-grains (see Figure 4c,f,i). The GND maps were found to capture the heterogeneity of deformation increase with the increase of cold-rolling reduction. In the 30%, 60% and 80% cold-rolled samples, the average GND density was 2.4 × 10^14^ m^−2^, 4.8 × 10^14^ m^−2^ and 11.29 × 10^14^ m^−2^, respectively. On the other hand, GND value in grains with γ-fiber orientations was higher than in α-fiber grains, i.e., orientation dependence. The GND value of the γ-fiber after 30% reduction was 2.9 × 10^14^ m^−2^; the α-fiber was 1.6 × 10^14^ m^−2^. After 60% reduction, there was a slight difference between γ-fiber and α-fiber with the values being 4.6 × 10^14^ m^−2^ and 2.9 × 10^14^ m^−2^, respectively. After 80% reduction, there was a significant difference between γ-fiber and α-fiber, which were 9.8 × 10^14^ m^−2^ and 6.5 × 10^14^ m^−2^, respectively.

**Figure 5:**
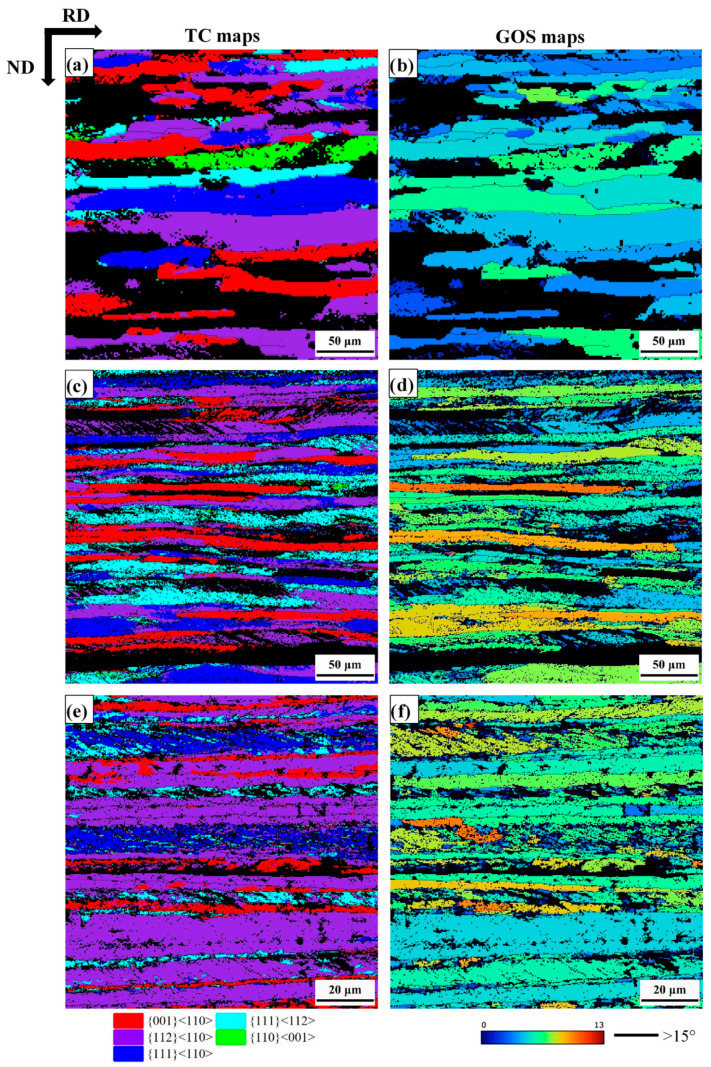
Texture components (TC) and GOS maps of (**a**,**b**) 30% cold -rolled, (**c**,**d**) 60% cold-rolled, (**e**,**f**) 80% cold-rolled.

**Figure 6:**
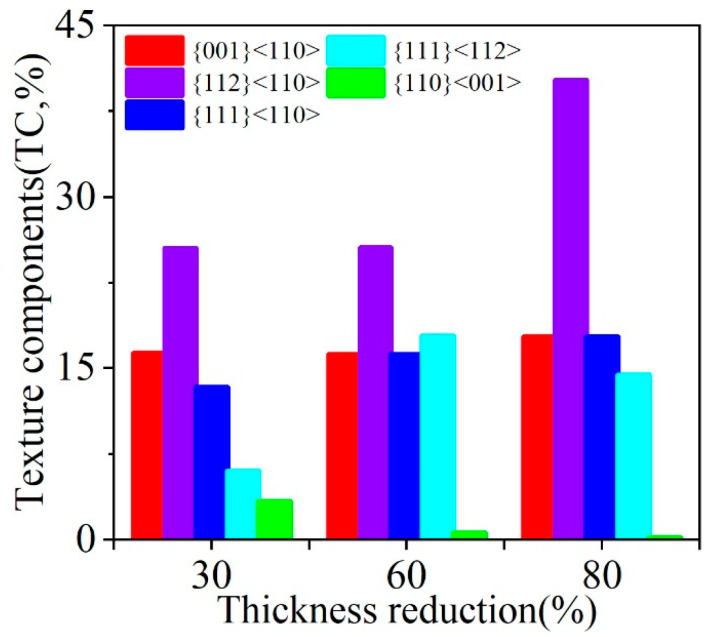
Fraction of TC as a function of rolling thickness reduction.

Figure 5 presents several texture component maps of cold-rolled samples and the grain orientation spread (GOS) maps of the main oriented grains. The texture composition of the sheets was a typical cold-rolled texture [46,47,48], with significant differences in their overall shape and internal structure of α-fiber grains ({001}<110>, {112}<110> and {111}<110>), and γ-fiber grains ({111}<110> and {111}<112>), as shown in Figure 5a,c,e. The α-fiber grains generally have a higher aspect ratio (i.e., more elongated in the RD and narrower in the ND) than γ-fiber grains. The mean thickness of the grains belonging to the α-fiber in the normal direction was 14.9 μm, 8.74 μm and 3.74 μm, while the mean thickness of the grains belonging to the γ-fiber was 16.33 μm, 11.86 μm and 5.97 μm for 30% reduction, 60% reduction and 80% reduction, respectively. There is no significant difference in mean thickness between {001}<110> and {112}<110> grains, or between {111}<110> and {111}<112>. The α-fiber grains have relatively little internal structure, while the γ-fiber grains were usually marked by a large number of bands (i.e., SBs). For a more in-depth analysis, Figure 5b,d,f presents GOS maps, which were obtained through EBSD analysis based on the local misorientation approach. As a reference for the reader, the value of GOS corresponds to the level of orientation change between each pixel in the grain and the average orientation of the grain. The main strain analysis tools reveal the most deformed grains by plotting their spatial distribution [49]. After 30% cold rolling, the average GOS value was 4.8°. When the reduction reached 60%, the average GOS value was 5.8° (55% of the grains with GOS value higher than 6°). It is indicated that the increase in true strain leads to an increase in the rotation vector. There are differences in the degree of rotation in different regions and orientations, resulting in a significant difference in orientation. With the further increase of true strain, the GOS value of 80% reduction was 4.8° (68% of the grains show a GOS value below 6°). The GOS value of {001}<110> and {111}<110> grains was more than other orientations. Figure 6 shows the fractions of {001}<110>, {112}<110>, {111}<110>, {111}<112> and {110}<001> in the 30%, 60% and 80% reduction samples. There is no obvious change in {001}<110> for different reductions, indicating that it is a stable orientation. At 80% reduction, the {112}<110> texture component accounts for 40.2%. As the reduction increases, the Goss texture component gradually decreases.

**Figure 7:**
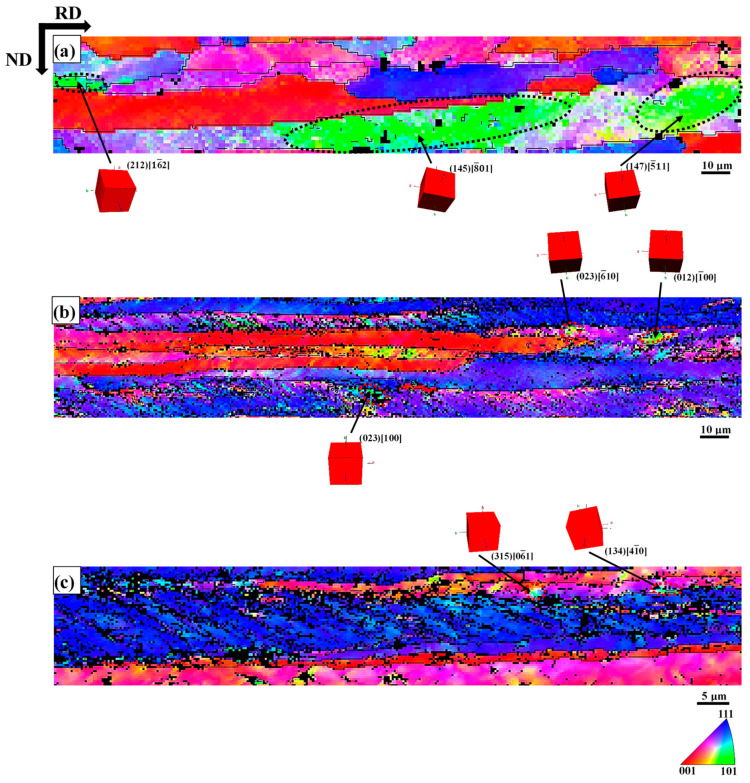
Part of {111}<112> grain in sample showing the Goss orientation of (**a**) 30% reduction, (**b**) 60% reduction, (**c**) 80% reduction.

Figure 7 illustrates the Goss orientation distribution near and inside the {111}<112> grains. At 30% cold-rolled reduction, the Goss orientation was composed of colony grains which distributed at the grain boundaries. The average length of the Goss oriented colonies in the RD direction was 80 μm, as shown in Figure 7a. At a reduction of 60%, the Goss orientation was composed of colonies and fragmental grains. The Goss colonies (with the average length of 18 μm in the RD direction) were mainly distributed at the grain boundary, and the fragmental Goss grains were distributed in the severe local deformation line (i.e., SBs) in Figure 7b. At 80% reduced, the Goss orientation was located in the grain boundaries and local deformation lines form in fragmental grains. With the increase of the reduction, the content of Goss orientation gradually decreases and the degree of fragmentation gradually increases.

**Figure 8:**
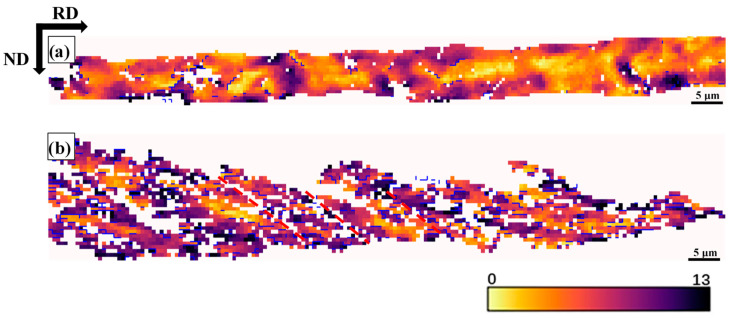
Grain Reference Orientation Deviation (GROD) angle in-grain taken from the 80% cold-rolled of (**a**) long range orientation in a {112}<110 >grain, (**b**) short range orientation in a {111}<121> grain. >6° disorientations appear as blue lines on the GROD maps. Red line marks the location of SBs.

**Figure 9:**
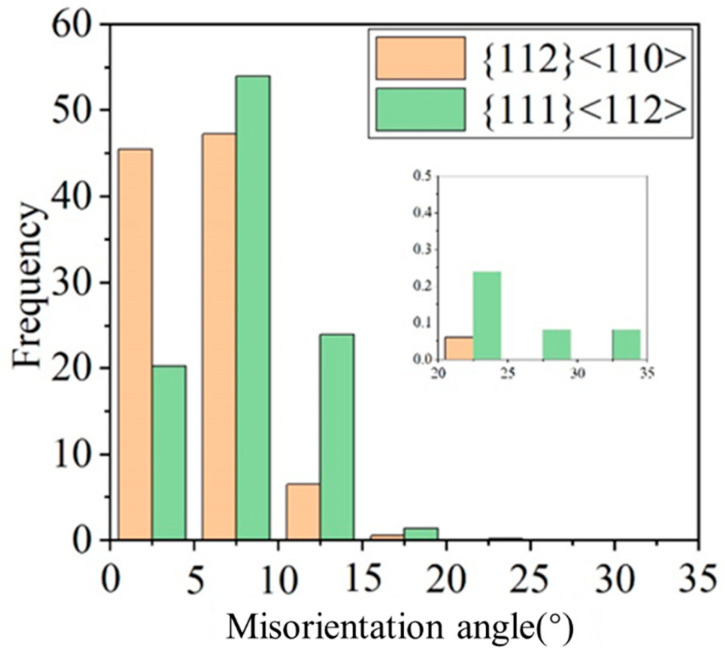
Distribution of misorientation angle pixel by pixel in grain.

Figure 8 reveals different in-grain orientation gradient behaviors with two grains taken from the 80% cold-rolled samples. It is well known that grains exhibit different tendencies to fragment in one way or another according to their orientation. The {112}<110> grain tends to develop long-range orientational measured parallel to the RD, but with few fragmented grains, while {111}<121> grain has short-range orientational but are much more fragmented [21,50]. In order to further quantify the variation in orientation gradient, the distribution of misorientation angle pixel-by-pixel in grain was calculated (see Figure 9). It is worth noting that the misorientation greater than critical value (10°~15°) may be more beneficial for recrystallization nucleation. The {111}<112> proportion of grain misorientation larger than 6° is far higher than {112}<110> grains.

### 3.2. Evolution of Texture During Cold Rolling

**Figure 10:**
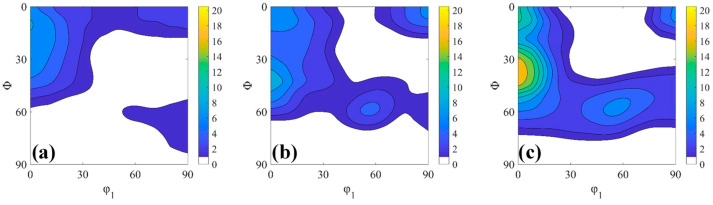
φ_2_ = 45° ODF sections showing measured cold-rolling textures deformed at reductions of (**a**) 30%, (**b**) 60%, (**c**) 80%.

The measured φ_2_ = 45° section of the ODF after cold rolling at 30%, 60% and 80% reductions are shown in Figure 10, and the ODF intensity is equal to a multiple of random intensity (i.e., random intensity = 1). As expected from prior work [5,25,51], the experimental textures exhibit strong α-fiber and weak γ-fiber. With the increase of cold-rolling reduction, both α-fiber and γ-fiber are gradually stronger (intensity increased). After 30% cold rolling, a weak discontinuous γ-fiber and an α-fiber (with stronger {117}<110> orientation) was obtained. With further increase in cold rolling to 60%, continuous γ-fiber and strong α-fiber was observed. The α-fiber had strong {223}<110> components, and γ-fiber had strong {111}<110> components. The ODF intensity of {223}<110> components became 17 times larger than random intensity, and the intensity for the γ-fiber components ranging from {111}<110> to {111}<112> increased at the rolling reduction of 80%.

### 3.3. Texture Simulation

**Figure 11:**
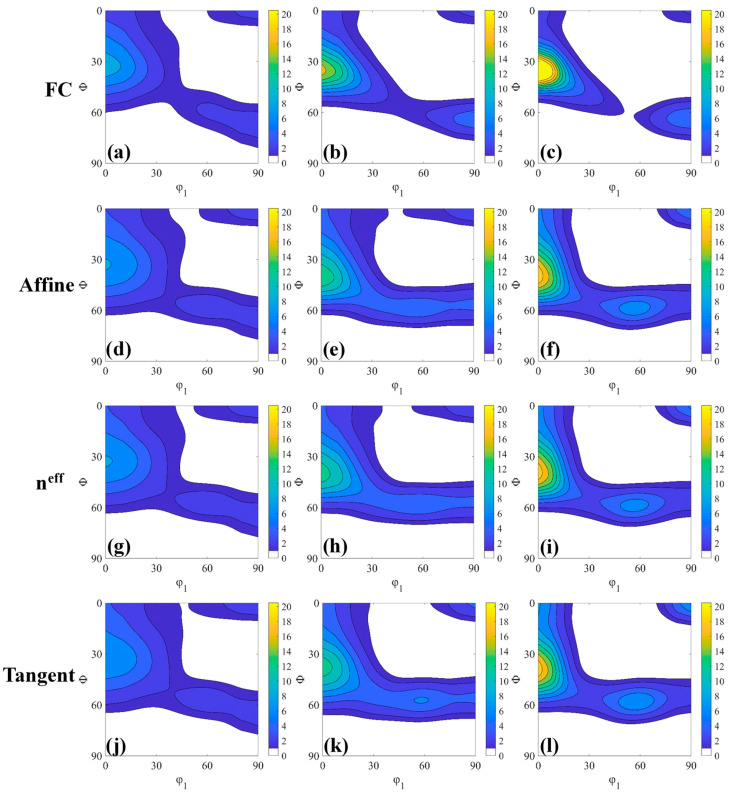
φ_2_ = 45° section of simulated ODF for 30% (**a**,**d**,**g**,**j**), 60% (**b**,**e**,**h**,**k**) and 80% (**c**,**f**,**i**,**l**) cold-rolled samples.

Figure 11 presents the φ_2_ = 45° section of the ODF showing cold-rolling samples deformed at 30%, 60% and 80% reductions calculated by the different inclusion-medium interaction models: FC, Affine, n^eff^ = 10 and Tangent. Figure 11a–c shows FC simulation for different deformed samples in which very strong α-fiber and weak and discontinues γ-fiber (at 80% cold-rolling reduction) were obtained. The other models qualitatively reproduce the correct first-order trends, i.e., formation of strong α-fiber and weak γ-fiber. Qualitatively, all inclusion-medium interaction models show reasonable matches, but the ODF intensity of different texture components may vary after simulation.

**Figure 12:**
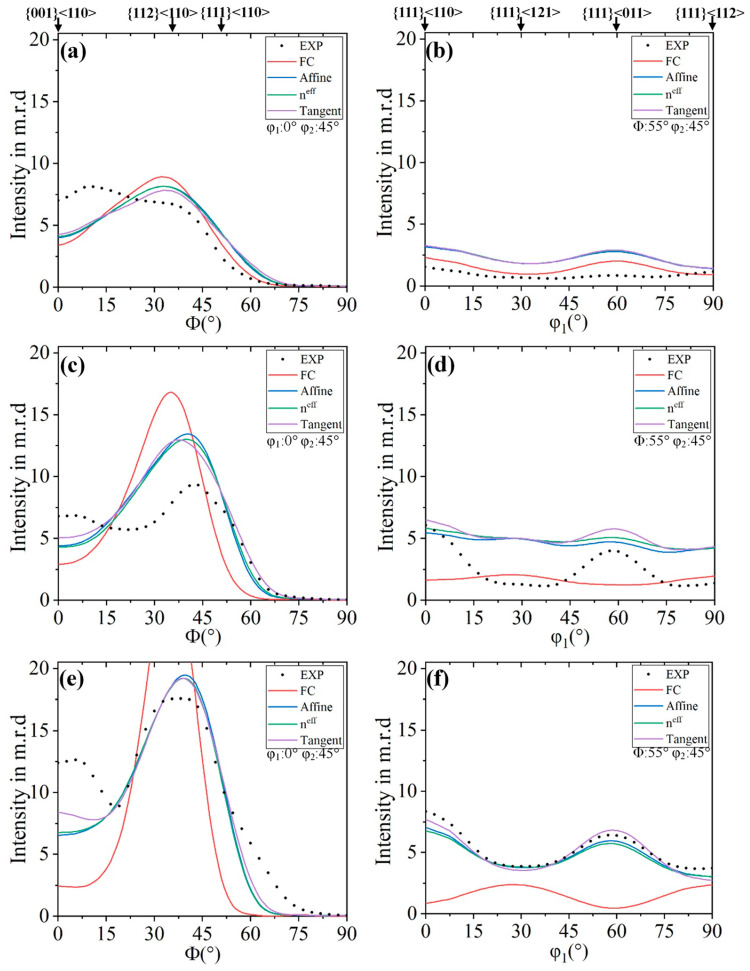
Fiber plots for experimental and VPSC models: (**a**,**b**) 30% reduction, (**c**,**d**) 60% reduction, (**e**,**f**) 80% reduction.

Figure 12 reveals the intensity m.r.d of texture components of different inclusion-medium interaction models along φ_1_ = 0° to φ_1_ = 90° in the φ_2_ = 45° ODF sections. At 30% reduction, all models have the same trend, and the overall predicted texture shows an overpredicted trend. The FC model has the most obvious deviation (in α-fiber), and the Tangent model has the smallest deviation. In α-fiber, the maximum value of the experimental texture component appears between {001}<110> and {112}<110>, and the maximum value of the predicted texture was near {112}<110>. In the γ-fiber, the experimental texture was very weak and there was no obvious peak, and the maximum value of the simulated texture was {111}<011>. At 60% reduction, except for the FC model, the others showed the same trend. In α-fiber, the highest peak of the experiment appears between {112}<110> and {111}<110>, and the maximum value of the predicted texture appears at the same position as the strongest peak of the experimental texture. In γ-fiber, the strongest peaks except FC appear near {111}<011>. The overprediction of the FC model was serious after the 80% reduction. Compared with the reduction of 60%, the degree of over-prediction of other models decreases overall. In γ-fiber, the Tangent model almost overlaps with the experimental texture, indicating that this model has more advantages in predicting severe plastic deformation texture. Considered comprehensively, although the Tangent model presents overpredictions, the overall trend and the difference in ODF intensity were within a reasonable range. It is notable that the Tangent model predicts the intensity of the γ-fiber well for up to 80% reduction. This γ-fiber is important in industrial applications owing to its strong effect on sheet formability via its high r-value [52,53]. As is well-know, even the full-field model poorly predicted the intensity of orientation. For example, Houtte et al. [16] and Bate et al. [26] reported a significant under-prediction and Delannay et al. [54] reported a CPFEM simulation significant overprediction of this texture component.

**Figure 13:**
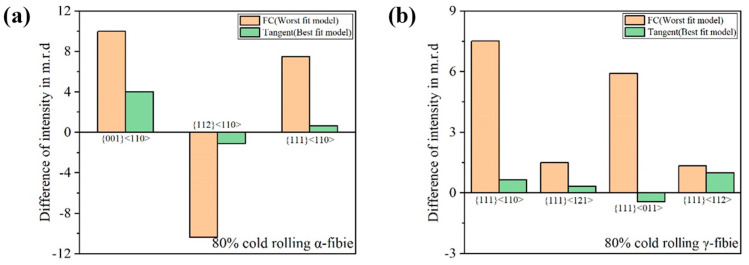
Variation of intensity in m.r.d of various texture components for (**a**) α-fiber and (**b**) γ-fiber of 80% cold-rolled samples for FC and tangent models.

Figure 13 shows the difference of intensity for various texture components between experiment and simulated textures after 80% cold rolling for the present samples. In the FC model, the {001}<110>, {111}<110> and {111}<011> were severely overestimated; the {112}<110> was greatly underestimated. It can be seen that the intensity difference of the Tangent model for all texture components is smaller than the FC model, which indicates that the Tangent model can predict cold-rolling texture reasonably better than the FC model.

## 4. Discussion

### 4.1. Effect of the Strain to Microstructure

In the current work, the banded structures inside the grains are referred to by the generic SBs. The SBs’ area has a higher average misorientation than the other microstructure areas, as shown in Figure 5b,d,f. SBs occurs inside a grain, but are not slip bands (since it has been reported in earlier studies [2,55], which are non-crystallographic). SBs are ±35° with respect to the RD. In this study, ferrite stainless steels undergo plastic deformation to form a grain structure with SBs, dividing large grains into smaller regions [35]. The formation of SBs is determined by the stress state and geometric constraints imposed during the deformation process, and their formation can be understood within the framework of the plastic instability criterion of Dillamore [56]. SBs in low carbon steel were formed in the high dislocation density area after severe plastic deformation [57]. These SBs are preferentially formed in the γ-fiber grains [42], which is consistent with observations (Figure 2). As the strain increases, the orientation with a higher Taylor factor (M) tends to rotate to a softer orientation and hence represents textural softening [55]. The γ-fiber has higher M, while α-fiber (except for {111}<110> which is an overlap) has lower M, which can explain the observed orientation dependence of SBs. In the rolling process of low-carbon steel, the SBs transfer the deformation from the material matrix through grain fragmenting (i.e., local material flow). The matrix does not rotate rapidly as it would otherwise, thereby weakening the deformation texture [40]. In warm rolled IF steel, transmission electron microscope (TEM) observations show that the change in micro-band density results in a combination of Goss and γ-fiber. In the recrystallization process of warm rolled IF steel, in-grain SBs play an important role in the formation of the nucleation of γ-fiber recrystallized grains [40].

During cold-rolling process observed with increasing cold-rolling reduction, there was a slight transition from HAGB to LAGB. Muñoz et al. [58] used EBSD to study the microscopic and substructure evolution of ARMCO iron through severe plastic deformation. HAGB converted to LAGB after the first pass, and LAGB converted to HAGB after the fifth pass. The transition from LAGB to HAGB can be associated with a dynamic recrystallization process, and the density of GND drops sharply [59]. In this study, the density of GND gradually increased with the increase of strain. In polycrystals, Ashby’s GND theory [12] indicates that the sudden change in the geometry of the slip system at grain boundary leads to the accumulation of GND density as dislocations are required to satisfy compatibility conditions and prevent the formation of overlapping grains. Figure 4c,f,i demonstrates that the grain boundary regions are the most likely places with the highest GND density in cold-rolled samples, which is consistent with the theory of Ashby. When reduction of deformation increases to 60%, the area (where the high GND density is located) is still at the grain boundary, but it spreads farther from the grain boundary to the inside of the grain. This trend still exists at a higher deformation level (i.e., 80% deformation), where the high GND density is located in the narrow band-shaped area at the grain boundary. With the increase of cold-rolling reduction, the difference between the GND density in the grain boundary region and the intragranular GND density gradually increases [11]. Nave et al. [13] reported the recrystallization texture of low carbon steel. It indicates that the Goss texture produced during the cold-rolling process will form a large amount of Goss texture after recrystallization. The Goss texture is mainly in the {111}<112> grain with SBs, which is consistent with observation (Figure 7). By increasing the rolling reduction, the volume fraction of Goss grains present in the microstructure can be further reduced (Figure 6). The deformation of α-fiber particles is relatively uniform (i.e., the GOS value is relatively low, as shown in Figure 5b,d,f), and γ-fiber has high unevenness in deformation [49]. Nave et al. [49] reported on the relationship between grain fragmentation and orientation in cold-rolled IF steel. The γ-fiber has a higher grain fragmentation than α-fiber. The γ-fiber has a short-range orientation gradient, and the α-fiber has a long-range orientation gradient (Figure 8). The work of Després et al. [21] supports the viewpoint.

### 4.2. Effect of the Interaction Model to Texture Prediction

The composition and intensity of the cold-rolled texture are determined by the initial texture, strain state and grain shape. In the cold-rolling experiment of BCC materials, two types of textures are usually seen, namely α-fiber and γ-fiber [11,50]. The low rolling reduction of low carbon steel (i.e., 30% cold-rolled) will produce incomplete γ-fiber (Figure 10a), the maximum intensity located at {001}<110> [60]. Wagner et al. [61] reported that cold-rolled low carbon steel showed the experimental and simulated ODF after VPSC simulation match very well during unidirectional rolling. The calculation was done using different hardening laws in the VPSC model, and the simulated texture does not show a significant deviation from the experimental data. Hence, the hardening parameters are not so important in the texture evolution of these lower-carbon steels by VPSC [61]. Adam et al. [62] used the VPSC simulated texture of BCC material (low carbon 1008 steel). Comparative experiments and simulated texture showed that the VPSC model qualitatively predicted the experimental texture evolution, but the intensity of the texture was overpredicted. The VPSC model tends to overall overpredict the texture, which leads to higher texture intensity. Compared with the experimental texture intensity, the simulation of the cold-rolled sample predicted strong α-fiber and weak γ-fiber, which qualitatively matched our experimental results. Takajo et al. [34] reported on the influence of VPSC simulation parameters on the texture development of low carbon steel after severe cold-rolling reduction (i.e., cold-rolled reduction of more than 90%). In the VPSC model, the same CRSS was used for the three bcc slip systems, and weak latent hardening and lower n value were used for texture simulation. Due to the large deformation, the evolution of the single grain shape was considered. They concluded that using an interaction model with an intermediate medium (n^eff^ = 10) can better reproduce the experimented texture [34]. Takajo et al. [22] reported that the simulation of different interaction models of ultra-low carbon steel with different reductions showed that the tangent model was suitable for 80% reduction. The FC model (at 80% reduction) shows strong α-fiber and lack of {001}<110> and γ-fiber ({111}<110>). The FC model is anisotropic latent hardening model, but the Eshelby tensor data did not converge well for the weak latent hardening model. The Affine, n^eff^ = 10 and Tangent model with isotropic hardening can better produce α-fiber and γ-fiber in Euler space of φ_2_ = 45° than the FC model. As the medium becomes compliant, the volume fraction of {112}<110> component of α-fiber tends to decrease and the volume fraction of {001}<110> and {111}<112> component increases when using the isotropic latent hardening model [36]. The Affine model was more suitable for considering the texture simulation of void materials [35]. In the present investigation, the tangent model matched the experimental texture reasonably well on the basis of texture intensity.

The mismatch in the texture intensity prediction between experiment and simulation can be due to many reasons. One possible reason is the existence of a large number of SBs in the microstructure. In the current work, the SBs formed during the cold rolling can be used as the interface of moving dislocations, and can also affect the rotation of grains and hence affected the development of texture. Madej et al. [63] reported that the effect of SBs on the texture development of metal materials in the plastic deformation process used the cellular automata-finite element multi-scale model. The appearance of the SBs affects the rotation rate of the grain and hence weakening the evolution of the texture during the cold-rolling process. Taken the influence of the SBs into account in the established model, the simulation results were in good agreement with the experimental results [64]. Jia et al. [65] reported that the crystal plasticity finite element texture simulation of plane strain compression α-brass. In addition to considering dislocations and twins, the SBs mechanism was incorporated into the constitutive model. The SBsrelated model produced weaker copper and S texture components and strong brass texture, indicating that the appearance of SBs would lead to a decrease in the lattice rotation rate. At a higher plastic strain (i.e., 80%), the influence of the SBs on the texture was more significant [65]. The cellular automata-finite element multi-scale model can better predict the texture because it combines the factors mentioned above. Whether this is applicable to the ferritic stainless steel in this investigation remains to be tested. However, the current results of this work remain novel and reliable.

## 5. Conclusions

In summary, the evolution of the microstructure and the deformation texture during cold rolling has been investigated by EBSD, and an interaction model that better reproduces ferritic stainless steels has been obtained by VPSC simulation. The following conclusions can be proposed:

- SBs were found to be the significant feature of the cold-rolling microstructure. With the increase of the cold-rolling reduction, the proportion of the SBs gradually increases. SBs were found to be orientation dependent, and γ-fiber grains tend to form SBs compared to other orientated grains. The appearance of SBs was explained based on the plastic instability criterion of Dillamore.
- The GND density increased with the increasing cold-rolling reduction. With the cold-rolling reduction increases, the difference between the grain boundary and intragranular GND density become larger. The phenomenon of GND were explained by Ashby’s theory. The value of GOS and grain fragmentation was found to be orientation dependent; α-fiber grains show a short-range orientation gradient and low GOS value, while γ-fiber grains show a long-range orientation gradient, severe grain fragmentation and high GOS value. With the increase of cold-rolling reduction, the volume fraction of Goss texture gradually decreases, and it is mainly located in {111}<112> grains.
- The cold-rolling samples showed strong α-fiber and weak γ-fiber. With the increase of the compliance of grain and HEM (except Affine), the intensity of the {001}<110> gradually increases, the intensity of the {112}<110> gradually decreases, and the intensity of γ-fiber gradually increases. Tangent VPSC interaction model was found to give reasonably good prediction of deformation texture development qualitatively and quantitatively for ferritic stainless steel.

## Figures and Tables

**Table 1:** Compositions of ferritic stainless steel (wt%).

C	Si	Mn	P	S	Cr	N	Ti	Nb	Fe
0.02	0.60	1.00	0.04	0.015	16–18	0.02	0.3	0.3	Bal.

**Table 2:** Typical inclusion and medium interaction models.

n^eff^	Interaction Model	Medium Stiffness
0	FC	stiff
10	n^eff^ = 10	Intermediate
20	Tangent	Compliant
→ ∞	Affine	Very compliant

## Data Availability

The original contributions presented in this study are included in the article. Further inquiries can be directed to the corresponding author.
